# On the function of the mammalian renal papilla and the peristalsis of the surrounding pelvis

**DOI:** 10.1111/j.1748-1716.2011.02261.x

**Published:** 2011-07

**Authors:** Bodil Schmidt-Nielsen, Bent Schmidt-Nielsen

**Affiliations:** 1 Department of Physiology, University of FloridaGainesville, FL, USA; 2 LexingtonMA, USA

**Keywords:** concentrating mechanism, kidney, peristalsis, renal papilla, renal pelvis, water removal

## Abstract

This is an informal personal review of the development over time of my ideas about the concentrating mechanism of the mammalian renal papilla. It had been observed that animals with a need to produce a concentrated urine have a long renal papilla. I saw the function of the long papilla in desert rodents as an elongation of the counter-current concentrating mechanism of the inner medulla. This model led me to overlook contrary evidence. For example, in many experiments, the final urine has a higher osmolality than that of the tissue at the tip of the papilla. In addition, we had observations of the peristalsis of the renal pelvis surrounding the papilla. The urine concentration falls if the peristalsis is stopped. I was wrong; together, these lines of evidence show that the renal papilla is not just an elongation of the inner medulla. We are left without a full explanation of the concentrating mechanism of the mammalian renal papilla. It is hoped that other researchers will tackle this interesting problem.

My interest in how some mammalian kidneys can produce highly concentrated urine goes back to 1947–1948 when I first worked with desert rodents in Arizona. As young research associates in Dr Laurence Irving's laboratory, my husband Knut and I were given the exciting problem of finding out how desert rodents manage to exist entirely without drinking water.

Working in a small desert laboratory in the Santa Rita Mountains in Arizona, we soon found that Kangaroo rats and several other desert rodents live and thrive on a diet of dry seeds only without access to drinking water. As I collected and analysed urine from the animals, I marvelled at these high concentrations of urea and salt in the samples. At that time I made up my mind to learn kidney physiology. Little was known about kidney function; in fact, so little was known that a specialist was quoted as saying: ‘All we really know about the kidney is that it makes urine.’

It struck me as very interesting that the kangaroo rats and several other desert rodents have very long renal papillae. At that time, my husband, Knut, got hold of a dissertation by Ivar Sperber in Sweden. Examining all of Sperber's drawings of various mammalian kidneys, I found that all animals with a long renal papilla were from arid environments (see Figure 1). Conversely, animals from moist habitats have a very short or no papilla at all. A few years later, the counter-current hypothesis was proposed and I assumed that the renal papilla could be looked at as an elongation of the inner medulla and therefore as a part of the counter-current system. According to the counter-current hypothesis, the urine concentration would follow the tissue concentration gradient. This is clearly not the case in the papilla.

**Figure 1:**
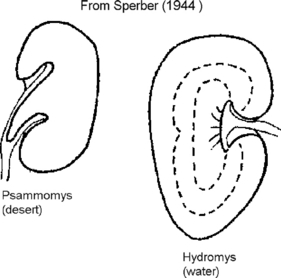
Contrasting kidney anatomy from dry and wet environments. Adapted from Sperber (1944) p. 317. The kidney from the desert rodent Psammomys has a long papilla, while the water dwelling Hydromys has no apparent papilla (Sperber 1944).

In 1964, Bruno Truniger and I found that urea concentration in the tissue stopped increasing at the border between the renal papilla and the upper part of the inner medulla. In the papilla, the concentration remained lower than in the urine (see Figure 2). The upper points in the figure are from animals on a high-protein diet and are the ones of interest here. A graduate student of mine, Susan Zell, did careful studies on a number of different desert rodents. In all cases, the urine osmolality far exceeded the tissue osmolality in any part of the papilla. Figure 3 shows an example of her results for the desert rodent Acomys. The concentration in the urine was more than 1000 mOsm higher than that in the tissue.

**Figure 2:**
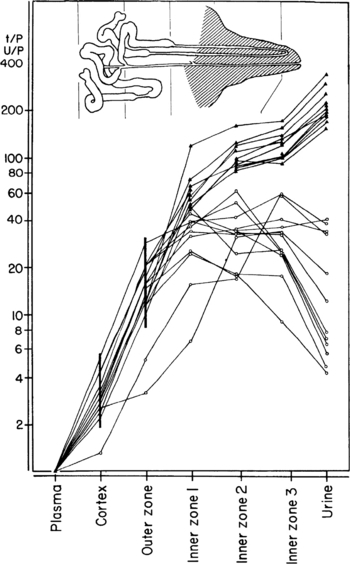
Distribution of urea in urine and renal tissue in rats on high-protein (solid triangles) and low-protein, high salt diet (open circles). U/P, urine-to-plasma ratio; t/P, tissue-to-plasma ratio (Truniger & Schmidt-Nielsen 1964).

**Figure 3:**
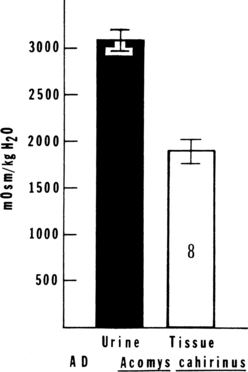
Average osmolality of bladder urine compared with average osmolality of papillary tissue from antidiuretic Acomys cahirinus killed by decapitation. Bladder urine was collected 30 min after spontaneous micturation (Zell 1973).

## The peristaltic mechanism

My interest in studying the peristalsis of the renal papilla stems from a chance observation. I had invited Karl Ullrich from Berlin to the US to collaborate with me. As we were preparing an animal for micro-puncture after we had injected lissamine green to colour the urine, we noticed that the peristalsis moved the blue-green urine in waves through the collecting ducts in the renal papilla. This intrigued me and I decided to study the peristalsis further. Another observation showed the importance of the peristalsis. In a collaborative study with Carl Gottschalk on the counter-current mechanism, his assistant would prepare antidiuretic animals for micro-puncture studies. For a better access, she would remove the pelvic wall surrounding the papilla. To our surprise, this procedure resulted in increased urine production with a lower osmolality in the prepared animals. At that point, we did not understand the meaning of the observation. Later, together with Bruce Graves, I started studies of the peristalsis.

There is a film clip of peristaltic waves in the supplemental materials.

**Figure 4:**
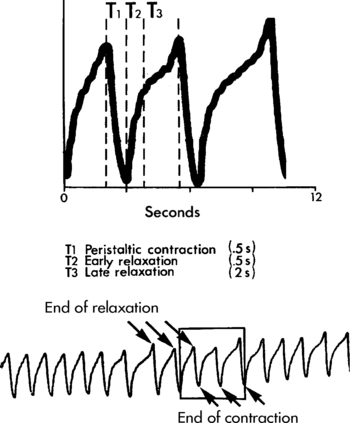
Fibre-optic recording of renal pelvic peristalsis in the hamster showing the phases of contraction and relaxation (Schmidt-Nielsen 1987).

**Figure 5:**
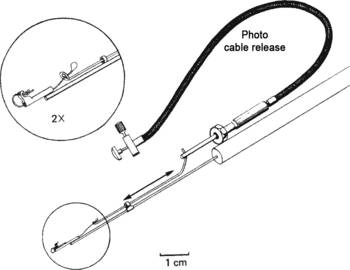
Drawing of the snare used to stop the peristalsis at a specific phase. The loop was placed loosely around the papilla about 1 mm from the tip (Schmidt-Nielsen & Graves 1982).

**Figure 6:**
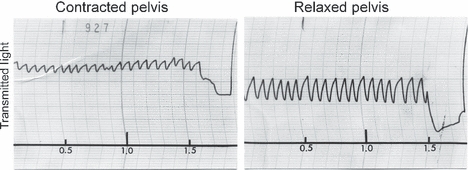
Fibre-optic recording of renal pelvic peristalsis showing the stopping of the peristalsis with the snare at contracted and relaxed phases (Schmidt-Nielsen & Graves 1982).

**Figure 7:**
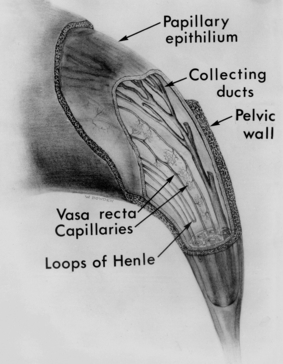
Schematic drawing of a hamster papilla with part of the pelvic wall and papillary epithelium cut away to show the structures (Schmidt-Nielsen & Graves 1982).

**Figure 8:**
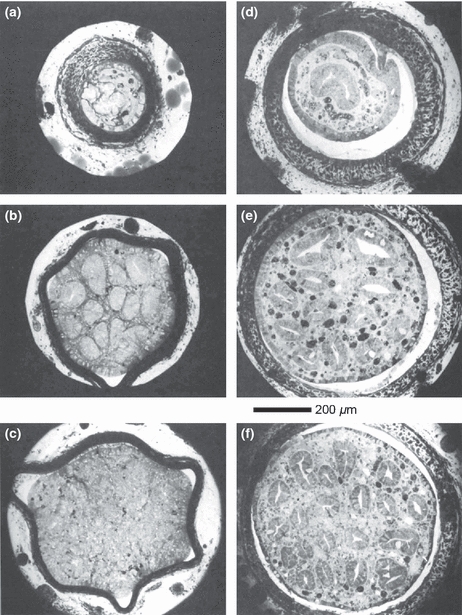
Cross sections from a hamster renal papilla with a contracted pelvis (left) and from a relaxed pelvis (right). The hamster was antidiuretic producing urine at 2300 mOsm. The two cross sections in each pair (a-d), (b-e), and (c-f) have the same number of collecting ducts (1, 12, and 20). Note the difference in appearance and thickness of the pelvic wall between the contracted and relaxed phases (Schmidt-Nielsen & Graves 1982).

**Figure 9:**
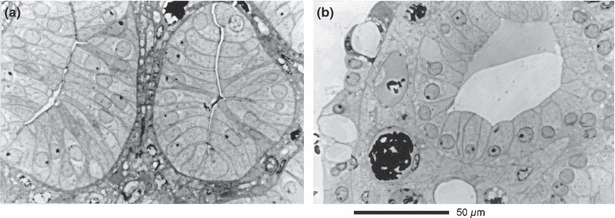
Higher magnification cross sections of the papilla 300 *μ*m from the tip showing the changes in collecting duct cells in the contracted (a) and relaxed (b) peristaltic phases (Schmidt-Nielsen & Graves 1982).

**Figure 10:**
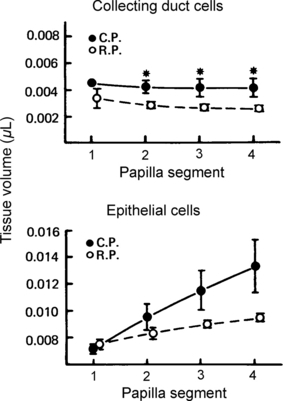
Computed volumes of the various structures in papillae stopped and fixed in the contracted phase (CP) and the relaxed phase (RP). The volumes are calculated in segments representing the volume between two papillary cross sections containing the following numbers of collecting ducts: ^#^1, 5–10; ^#^2, 10–15; ^#^3, 15–20; ^#^4, 20–25. Each point represents the mean ± SEM of four papillae (Schmidt-Nielsen & Graves 1982).

To track the peristalsis, we trans-illuminated the papilla with a fibre-optic light and recorded the light coming through the papilla on a chart recorder (see Figure 4). You can see the peristaltic contraction and relaxation phases clearly on the figure. To study the effect of the peristalsis on the renal papilla, we wanted to stop the action. Using the snare shown in Figure 5, we stopped the peristalsis at relaxed and contracted phases, as indicated in Figure 6, and rapidly fixed the tissue by pouring fixative directly on the preparation. Figure 7 is a schematic diagram of the papilla. As you can see, the entire papilla including the papillary epithelium is surrounded by the contractile pelvic wall. On the left of Figure 8 are cross sections of a contracted papilla and on the right are sections of a relaxed papilla (cf. Fig. 6). You can clearly see the contracted pelvic wall as a dark outline on the left. Figure 9 shows higher magnification cross sections 300 *μ*m from the tip. We counted the number of collecting ducts in each cross section to define the segments of the papilla. We measured cell volumes by cutting out the relevant portions of micrographs of serial sections and weighing them. Figure 10 shows our results for cell volumes along the length of relaxed and contracted papilla. As you can see, epithelial cell volume is actually greater in the contracted papilla. And for the collecting duct cells, the effect is stronger and increases along the length of the papilla.

To study the physiological effect of the peristalsis, Bruce Graves and I performed experiments on pelvic paralysis or removal of the pelvis. Similar experiments were also performed in Mark Knepper's laboratory (Pruitt *et al.* 2006). One kidney in the live rat was treated and the other was sham operated as a control. Either paralysis with Xylocaine or surgical removal of the pelvis significantly reduces the papillary osmolality.

In 2002, together with an artist in Gainesville, I created an animated cartoon of my proposed model of the effect of the peristalsis on the function of the renal papilla.

A film clip narrated by myself at that time is available in the supplemental materials.

## Hypothesis on how the renal pelvic peristaltic pumping of the papilla might contribute to the concentrating mechanism

[This section is a mildly edited transcript of the video. It should be noted that the hypothesis shown in the video and in the following transcript is incomplete. In particular, an accounting of the forces moving water is far from complete. I suggest that this is a fertile area for future research].

On the basis of experimental findings by a number of investigators including us, I shall now present a hypothesis and an animation on how the renal pelvic peristaltic pumping of the papilla might contribute to the concentrating mechanism. The model deals only with mammals with a relatively long papilla. The highest degree of urinary concentration is found in mammals with a long renal papilla. In these, the peristalsis has the strongest effect on the papilla. I suggest that the kidney papilla works as a pump through alternating positive and negative pressures generated by the peristaltic contractions of the pelvic wall.

1. Water moves into the collecting duct cells as a result of the small positive hydrostatic pressure on the walls of the cells, generated by the peristaltic wave pushing the fluid through the collecting ducts.
2. Fluid moves out of the cells as a result of the negative pressure generated by the elastic forces, which expand the papilla during rebound.
3. Fluid is removed from the interstitium by the vasa recta, which contain no blood at the time the fluid enters.
4. The animated model shows a hamster papilla in cross section and then in a longitudinal section.
5. To concentrate the urine in the collecting ducts, water must be removed from the collecting duct fluid.
6. Partly, this water removal is caused by the accumulation of solutes in the papillary interstitium.

The model presented here tries to explain how the hydrostatic pressure generated by the pelvic wall peristalsis could contribute to the removal of water from the collecting duct urine. It does not deal with the solute. Water movements through a membrane result from the difference in water potential in the two compartments separated by the membrane. Water potential is decreased by solutes in solution and increased by hydrostatic pressure. Water moves through water channels (aquaporins). As shown by Mark Knepper and his colleagues (Nielsen *et al.* 2002), water leaves the collecting ducts lumen through the aquaporin water channels in the plasma membranes of the collecting ducts cells. Aquaporin-2, the antidiuretic hormone-sensitive water channel, is present in the apical membrane of the collecting duct cells.

Water molecules move through the aquaporins by single-file diffusion without entrainment of solutes.

Urea moves by diffusion through urea transporters. Water can leave the collecting duct cells through the water channels, aquaporins three and four, which also permit solutes to pass through.

In the animated model, the sequence of the events occurring in the papilla is shown at low urine flow.

1. Late relaxation lasts about 2 s. The shape of the papilla remains normal. The collecting ducts open as urine flows into them from above during the last second of this period. Vasa recta remain open and blood flows through them. Loops of Henle remain open as fluid continues to flow through them.
2. Peristaltic contraction lasts about 0.5 s. In front of the contraction, the collecting duct fluid is pushed through the ducts at a velocity greater than the velocity with which the fluid is formed, thus creating an increment in the pressure on the wall of the ducts.
3. About half of the fluid in the terminal collecting duct is absorbed. Our findings indicate that the collecting duct cell volume in the terminal collecting ducts is increased by approximately the amount of fluid volume absorbed from the collecting duct lumen during each peristaltic contraction.
4. This suggests that the reabsorbed water enters the collecting duct cells. Collecting ducts close behind the urine bolus as the papilla is stretched becoming longer and narrower. The vasa recta close and blood flow stops.
5. Some blood moves retrograde, some down towards the tip of the papilla. The loops of Henle close as fluid is pushed both retrograde and towards the tip.
6. During rebound, the papilla becomes shorter and broader. Collecting ducts are still closed, but water moves out of the cells into the interstitium due to negative hydrostatic pressure generated by the elastic properties at the interstitial matrix.
7. Ascending vasa recta are tethered to other structures and will open as tissue expands during rebound permitting water to enter the vasa recta. At this point in time, there is no blood in either the ascending or descending vasa recta. Loops of Henle, descending as well as ascending, are still empty.
8. Early relaxation lasts about 1 s. The papilla resumes its original shape. Collecting ducts are still empty and closed because the urine has not reached them yet. Vasa recta, first the descending then the ascending, are filled with blood pushing the column of water that had entered the ascending vasa recta towards the cortex. Loops of Henle are also filled with fluid.

## Conclusion

The peristaltic mechanism proposed above is currently incomplete. Our knowledge of the papillary concentrating mechanism is insufficient to explain its action. However, the data clearly show that the papilla is an important part of the overall concentrating mechanism.

The mammalian kidney has two concentrating mechanisms. One is the counter-current mechanism that establishes a concentration gradient in the renal tissue increasing from cortex to the inner medulla by the addition of solutes to the tissue. The other less well understood concentrating mechanism is based on removal of water from the collecting duct fluid to conserve water for the organism. The data have shown that the increase in urine osmolality in the renal papilla is due to water removal. Fluid of a much lower osmolality than that of the final urine is removed from the collecting ducts. The forces responsible for this water movement are not yet understood. For example, Zell's observations (Fig. 3) of urine osmolalities more than 1000 mOsm kg^−1^ greater than that of the tissue would suggest osmotic pressure differences greater than 25 bar. Such pressures are far greater than can reasonably be generated by the peristalsis. The peristalsis of the pelvic wall enclosing the papilla is nevertheless an essential component of the concentrating mechanism. If peristalsis is disabled by removal of the pelvic wall or by paralysis with Xylocaine, the concentrating ability is significantly reduced (Pruitt *et al.* 2006).

At the Arizona Symposium (Dantzler 2006), it was suggested anecdotally by Bill Dantzler that the renal papilla functions differently from the inner medulla. It is not just an elongation of the inner medulla. This comment triggered a rethinking of my ideas. Many of my previous findings strongly support such a proposal. Knepper *et al.* (2003) have written an excellent review of the concentrating mechanism of the renal inner medulla. They have also shown that aquaporins are present in the papilla. These presumably play an important role in the concentrating mechanism.

Some insects also have a remarkable concentrating ability in their excretory organs. Such insects might use a related mechanism. When I gave the August Krogh lecture at the American Physiological Society (Schmidt-Nielsen 1995), I compared the concentrating mechanism in insects with that of desert rodents. Insects have amazing powers of conserving water. There again, we do not fully understand the mechanism. However, the insects have a mechanically strong chitinous tissue that is muscular and packed with mitochondria.

We are left with many problems yet to be solved. My father would quote Piet Hein saying, ‘Problems worthy of attack prove their worth by hitting back.’

## Supporting Information

**Video Clip S1.** Syrian hamster kidney papilla. Infused with lissamine green. Showing waves of green colored urine pushed by the peristalsis.

**Video Clip S2.** A cartoon animation showing a proposed model of the concentrating mechanism of the renal papilla under peristalsis. The animated model shows a hamster papilla in cross section and then in a longitudinal section.
